# Cariogenicity of *Candida albicans* of distinct genotypes among 3-5-year-old Uygur children in Kashgar, Chi*na-* a case-control study

**DOI:** 10.1186/s12903-018-0658-4

**Published:** 2018-12-05

**Authors:** Wanting Zhang, Yan Li, Jing Lin, Aynur Abduryim, Jin Zhao

**Affiliations:** 1 Department of Endodontics, The First Affiliated Hospital of Xinjiang Medical University, Urumqi, Xinjiang, 830054 China; 2 Xinjiang Kashgar First people’s Hospital, Xinjiang, 844000 China

**Keywords:** Early childhood caries, *Candida albicans*, Ethnicity, Cariogenicity, Genotype

## Abstract

**Background:**

In recent years, the opportunistic fungus C.albicans has been linked to ECC.It is important to investigate the relationship between the distribution of C.albicans and early childhood caries (ECC) in 3–5-year-old Uygur and Han children in Kashgar, and the role of C.albicans of distinct genotypes in caries.

**Methods:**

Two hundred fifty-six Uygur and 141 Han children were enrolled in the study. The identified C.albicans isolates were genotyped based on 25S rDNA Polymerase Chain Reaction(PCR) amplification, and their acidogenicity, aciduricity, and adhesiveness were examined. Moreover, secreted aspartic protease (Sap) activity and SAP1–5 transcriptional levels were compared in the ECC and caries-free (CF) groups of Uygur children.

**Results:**

C.albicans detection rate was significantly higher in Uygur children than in Han children (44. 5% vs. 31. 2%; χ2= 6.739, *P* = 0.009);the A genotype was dominant in Uygur and Han children with ECC, the C genotype was dominant in Uygur CF children(*P* = 0.022). C.albicans acidogenicity and growth were more pronounced in the Uygur ECC group than in CF group, especially at pH 4.0 and 4. 5(pH 4.0, *P* = 0.012; pH 4. 5, *P* = 0.029); the average ratios of glass-wall adhesion and the Sap activity was higher in ECC group than in CF group(*P* < 0.01), and SAP2(*P* < 0.001) and SAP5(*P* = 0.001) were expressed more robustly in ECC group.

**Conclusions:**

The strong acidogenicity and aciduricity, Sap activity, and high SAP2 and SAP5 expression might be closely associated with ECC. C.albicans potentially plays a key role in the progression of caries, which most readily affects genotype A carriers and could be attributed to person-to-person environmental variation.

## Background

Early childhood caries (ECC) is the most common disease in children [1]. Numerous epidemiological studies have demonstrated that the prevalence of ECC is lower in developed countries than in developing countries with low socioeconomic status [2, 3]. Hence, children in underdeveloped regions might be at a higher risk of caries, and stringent measures must be employed to treat them [4]. ECC is recognized as caused by a combination of diverse microorganisms and multiple factors. The research on ECC-associated microorganisms has mainly focused on the bacteria *Streptococcus mutans*, *Lactobacillus*, and *Actinomyces* [5]. In recent years, the opportunistic fungus *C.albicans* has also been linked to ECC: it is detected in the saliva, plaque, and caries tissues, and the detection rate is markedly higher in children with ECC than in caries-free (CF) [6].

Dental plaque, a microecological environment, where the microbes survive closely attached to the tooth surface, slows the local diffusion of saliva, enabling a continuous partial activity of acids; once the cariogenic pH (5. 4, 5. 5) is reached, enamel demineralization occurs, with caries formation [7]. *C.albicans* ferments various sugars and produces acids; it can reduce the pH of a sugary broth from 7.0 to 3. 5, potentially leading to tooth demineralization [8]. A key *C.albicans* virulence factor is secreted aspartic protease(Sap; gene:*SAP*), it can promotes *C.albicans* adhesion to tooth surface [9]. The probability and intensity of the disease caused by *C.albicans* of distinct genotypes are different, which may correlate with the ethnic and regional disparities between different geographical areas, and *C.albicans* strains harboured by different groups of people harbour distinct genes [10]. Until now, only a few studies have examined *C.albicans* gene polymorphism in relation to the underlying mechanism of caries, and fewer still have investigated the influence of ethnic and geographic factors.

Xinjiang province is located in Northwestern China, with the Uygur people as the predominant ethnic minority group. The incidence of ECC among 3–5-year-olds in Kashgar city is 74.58% [11], which is markedly higher than the average level of China. This suggested that these children are particularly ECC-prone. The Uygur account for 91.92% of the total population in Kashgar; Kashgar’s history, geography, and local customs render the city a isolated area with a unique representation of the Chinese population. Hence, in the current study, we investigated *C.albicans* distribution, and the relationship between *C.albicans* and ECC in 3–5-year-old children in Kashgar. We also examined the role of *C.albicans* of distinct genotypes in the mechanism of caries development, providing a theoretical reference point for dental caries prevention.

## Methods

### Study participants

This study was approved by the First Affiliated Hospital of Xinjiang Medical Ethics Committee (ethical review number 20150214–162), and the local heath administrative departments. We used the following sample size estimation formula [10, 12]:

$$ \mathrm{N}={\left({\mathrm{Z}}_{1\hbox{-} \upalpha /2}+{\mathrm{Z}}_{1\hbox{-} \upbeta}\right)}^2\left[{\mathrm{p}}_1\left(1\hbox{-} {\mathrm{p}}_1\right)+{\mathrm{p}}_2\left(1\hbox{-} {\mathrm{p}}_2\right)\right]/{\updelta}^2={\left(1.96+1.28\right)}^2\left[0.5\ast \left(1\hbox{-} 0.5\right)+0.75\ast \left(1\hbox{-} 0.75\right)\right]/{\left(0.75\hbox{-} 0.5\right)}^2=73.5. $$

This yielded a minimum total sample size of 296.

After applying the stratified cluster-sampling method, 397 3–5-year-old children were enrolled in the study. The children were healthy; presented no systemic, hereditary, or mucous diseases; did not wear orthodontic appliances; and had not received any antibiotic drugs or injections within 30 d prior to sampling. Written consent was obtained from the parents (legal guardians) before sampling.

### Oral examination and specimen collection

Two trained oral physicians checked 20 teeth of each child. The children were divided into ECC and CF groups. ECC is usually defined as the presence of one or more decayed (both noncavitated or cavitated lesions), missing (due to caries), or filled tooth surfaces in any primary tooth in a child under the age of six (by AAPD). The inter-examiner reproducibility was 0.83, and the intra-examiner reproducibility was 0.81.The children rinsed their mouths before sampling; the dental plaques were collected by a sterilized caries excavator from the third cervical on the buccal side of the first maxilla and the mandibular deciduous molar, and one third lips of the upper anterior teeth [10]. The samples were placed in 1.5 mL sterile Sabouraud liquid medium (SDB)maintained 4°C, and returned to the laboratory within 2 h.

### Isolation and purification of *C.albicans*

Each sample (20 μL) was used to inoculate on CHROMagar (CAC) medium and cultured aerobically at 37 °C, the colonies were evaluated after 24–72 h. Emerald green single colonies were picked and streaked on another CAC plate, the procedure was repeated three times.

### Identification of *C.albicans*

The morphology of cells from *C.albicans* colonies were examined under a microscope after Gram staining. For germ-tube experiments, microbial suspensions and bovine serum were mixed, placed in a moist dish, incubated at 37 °C; samples were stained every 60 min, and examined cell morphology.

*C. albicans* was also identified by PCR. Fungal DNA was extracted using the Biospin DNA extraction kit. The following primers were used: ITS1 (5′-GGAAGTAAAAGTCGTAACAAGG-3′) and ITS2 (5′-GCTGCGTTCTTCATCGATGC-3′). The PCR mixture contained 2×Easy Taq PCR supermix(10 μL), 10 μmol·L^-1^ of forward and reverse primers (0. 5 μL each), DNA template (2.0 μL), and ddH_2_O (7.0 μL). The cycling conditions were as follows: 95 °C hot-start, 5 min; followed by 40 cycles of 95 °C for 30 s, 50 °C for 30 s, 72 °C for 30 s; and 72 °C for 10 min. The PCR products were imaged using a UV gel scanner after agarose electrophoresis.

Following identification, 20 *C.albicans* strains were randomly selected from ECC and CF Uygur children, and from ECC Han children for subsequent genotyping.

### 25S rDNA PCR-based *C. albicans* genotyping

The 25S rDNA PCR amplification mixture contained 2× Easy Taq PCR supermix (15 μL), 10 μmol·L^-1^ of forward and reverse primers (1.0 μL of each), DNA template (2. 5 μL), and ddH_2_O (10. 5 μL); the following primers were used: CA-INT-L (5′-ATAAGGGAAGTCGGCAAAATAGATCCGTAA-3′) and CA-INT-R (5′-CCTTGGCTGTGGTTTCGCTAGATAGTAGAT-3′). The cycling conditions were as followed: 94 °C hot-start, 3 min; 30 cycles of denaturation at 94 °C for 1 min, annealing at 67 °C for 1 min, extension at 72 °C for 2. 5 min; and final extension at 72 °C, 7 min. The different genotypes were determined based on the sizes of amplification product bands.

### Acidogenicity and aciduricity test

SDB media containing different glucose concentrations (0.01–0. 2 mol·L^-1^) and at different pH values(4.0–7.0) were prepared. Microbial suspensions were adjusted to OD_540_= 1.0, inoculated into SDB [1:10 (*v*/v) ratio], and aerobically cultured at 37 °C for 48 h. Next, each suspension was centrifuged (15 min, 5000 rpm × *g*, 4 °C), and the supernatant was transferred to another tube. The pH of the supernatant (terminal pH) was measured and the pH change calculated [ΔpH = pH(initial)–pH(terminal)]. The collected *C.albicans* pellet was diluted in 4 mL of sterile saline, vortex-mixed, and shocked for 25 s; OD_540_ was then measured, with the sterile saline as a blank. Each sample was tested three times and the results were averaged.

### Adhesiveness test

Microbial suspensions (1 mL; OD_540_= 1.0) were added to SDB (1 mL), the tubes tilted at 30°, and cultured aerobically at 37 °C, overnight. Next, the tube containing the suspension (tube no. 1) was gently rotated three times. The content was transferred to a clean tube (tube no. 2); 6 mL of PBS was placed in tube 1, which was again gently rotated three times. The tube contents were transferred to a third tube (tube no. 3) [13]. Tubes no. 2 and 3 were centrifuged (10,000 rpm, 10 min), and the supernatant removed; 6 mL of PBS was placed in tubes no. 1–3, and the contents mixed evenly. Next, OD_540_ of tube no. 1–3 contents was measured. The microbial adhesion ratio was calculated as follows: adhesion ratio = OD1/(OD1 + OD2 + OD3) × 100%. Each sample was evaluated three times and the data were averaged.

### *C. albicans* sap activity

For the YNB-BSA-agar method [14]; *C.albicans* suspensions were adjusted to 10^6^ CFU·mL^-,1^ 5 μL drops were pipetted onto YNB-BSA agar in triplicate, and the plates incubated aerobically at 37 °C for 72 h. The protease activity (Pa) was calculated as the ratio of colony diameter to colony-and-halo diameter.

For the MTT assay with BSA as substrate, microbial suspensions were diluted to 10^6^ CFU·mL^-1^ and centrifuged (1500 rpm, 4 °C, 10 min); the supernatants were added to 0. 1 mol·L^-1^ of citrate buffer containing 0. 2% BSA, and incubated aerobically at 37 °C for 30 min. The reaction was terminated by the addition of 10% (*w*/*v*) trichloroacetic acid; the samples were then centrifuged (1300 rpm, 4 °C, 30 min), and OD_280_ of the supernatant was measured. Next, the microbial suspension was transferred, in triplicate, to 96-well plates (200 μL per well); 20 μL of MTT was added to each well, and the plates were incubated in the dark (37 °C, 4 h). Finally, the supernatant was collected, mixed with dimethyl sulphoxide (150 μL), and agitated gently for 10 min; OD_490_ of each sample was then measured. The increase in absorbance corresponded to Sap activity (calculated as the OD_280_/OD_490_ ratio) [15].

### *SAP1–*5 gene expression

Total RNA was extracted from *C.albicans* cells and then reverse-transcribed to cDNA using a cDNA synthesis kit. RT-PCR quantification of *SAP1–*5 expression was performed in 20 μL reaction mixtures containing cDNA template (2 μL), 10 μmol·L^-1^ of forward and reverse primers (0. 5 μL each;Table 1), 5× SYBR Green I PCR buffer (10 μL), and ddH_2_O (7.0 μL). The cycling conditions were as follows: initial denaturation at 95 °C for 30 s; followed by 40 cycles of denaturation at 95 °C for 5 s, elongation at 60 °C for 30 s. The results were then analysed.

**Table 1:** Primers for *SAP1–*5 and *GAPDH*

Primer name	Primer sequence	Fragment length (bp)
SAP1	F TCAATCAATTTACTCTTCCATTTCTAACA	161
	RCCAGTAGCATTAACAGGAGTTTTAATGACA	
SAP2	F TGGATTTGGTGGTGTTTCGA	108
	R CCACCGGCTTCATTGGTTT	
SAP3	F CCTTCTCTAAAATTATGGATTGGAAC	231
	R TTGATTTCACCTTGGGGACCAGTAACATTT	
SAP4	F CATTCATTCCTTTAATACCGACTATC	156
	R GGTAACAAACCCTGTAGATCTTTTAAC	
SAP5	F TGGTGGTATTGACAAGGCCA	107
	R TTCGTCCCCTAACATTGACAGAT	
GAPDH	F TTGACGGTCCATCCCACAA	103
	R GGAATAACCTTACCAACGGCTTT	

### Statistical analysis

Data were analysed using SPSS 21.0. For categorical data, χ^2^ test was used (α = 0.05, two-sided). For continuous data,when normality and homogeneity were detected, *t* test or the nonparametric rank-sum test was used. Finally, factorial analysis was employed to evaluate data from the following experiments: acidogenicity and aciduricity (ΔpH, ΔOD), adhesion ratio, Sap activity, and *SAP1–*5 expression in *C.albicans* isolates of different genotypes. The differences were considered statistically significant when *P*<0.05.

## Results

### Distribution of *C.albicans* in the dental plaque of Han and Uygur children

Based on the proportion of local population distribution, 256 Uygur and 141 Han children were enrolled in the study. *C.albicans* detection rate was significantly higher in Uygur children than in Han(44. 5% vs. 31. 2%; χ^2^= 6.739, *P* = 0.009), and in the ECC group than in the CF group of Uygur children (52. 3% vs. 27. 5%; χ^2^= 13.665, *P* < 0.001) and in the ECC group than in the CF group of Han children (41.0% vs. 19.0%; χ^2^ = 7.842, *P* = 0.005). Among the Uygur children, the detection rate of *C.albicans* was significantly higher in male than in female children(51. 2% vs. 37.8%; χ^2^= 4.630, *P* = 0.031)(Table 2).

**Table 2:** The distribution of *C.albicans* among Uygur and Han children

Ethnicity	Caries		P	Gender		P	Total	P
	ECC (%)	CF (%)		Male	Female			
Uygur	92/176 (52. 3)	22/80 (27. 5)	< 0.001	66/129 (51. 2)	48/127 (37.8)	0.031	114/256 (44. 5)	0.009
Han	32/78 (41.0)	12/63 (19.0)	0.005	21/73 (28.8)	23/68 (33.8)	0.517	44/141 (31. 2)	
Total	124/254 (48.8)	34/143 (23.8)	< 0.001	87/202 (43. 1)	71/195 (36. 4)	0.175	158/397 (39.8)	

*ECC* early childhood caries, CF caries-free

### *C. albicans* identification

**Fig. 1:**
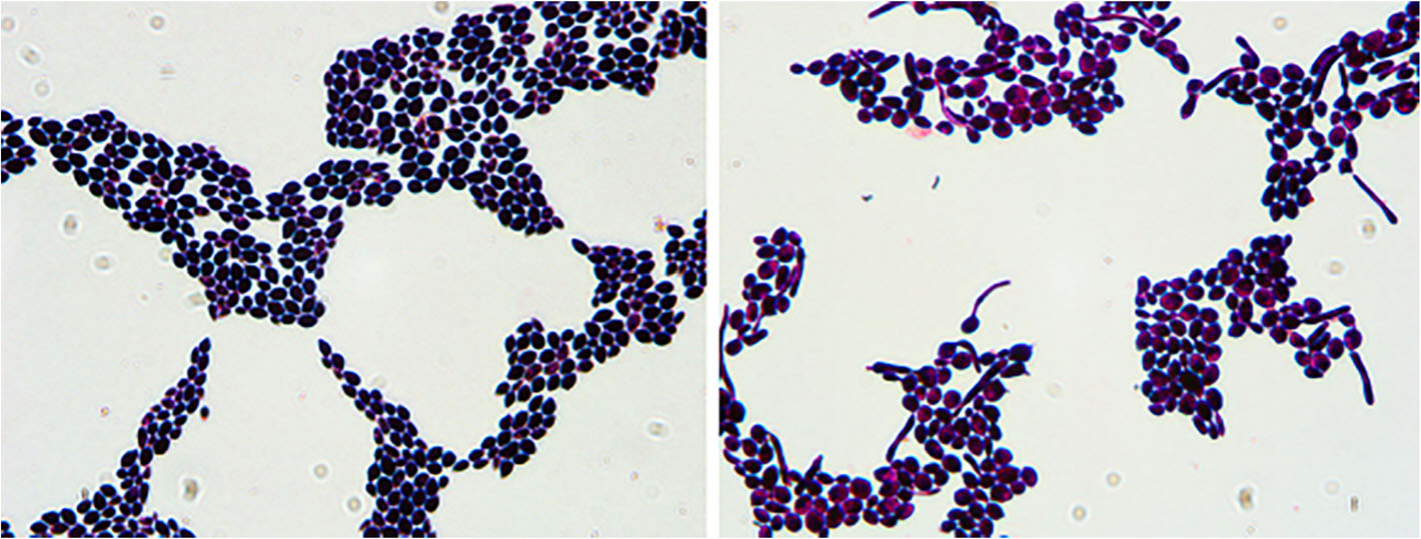
Gram staining of C.albicans (× 100; left panel) and germ-tube test (× 100;right panel)

**Fig. 2:**
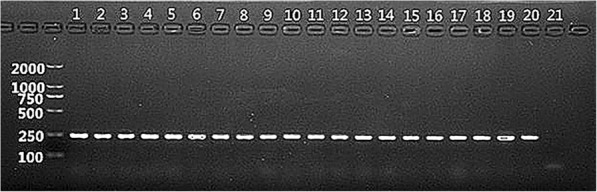
PCR-based identification of C.albicans. A ca. 250-bp band was detected in all isolates after amplification(molecular marker is shown on the left). Lane 1: standard C.albicans strain ATCC 90028; lanes 2–10: C.albicans from Uygur children with early childhood caries (ECC); lanes 11–20: *C. albicans* from Uygur caries-free (CF) children; lane 21: negative control

After 24 h culture on CAC plates, the *C.albicans* clinical isolates formed white or light green, flat, and round colonies; after 48 h, the colonies appeared emerald green, and had a smooth surface. Gram-positive *C.albicans* cells were round and scattered (Fig. 1, left). In the germ-tube test, the germinated spores of *C.albicans* clinical isolates were circular, with slender hyphae (Fig. 1, right). PCR analysis confirmed that all suspected clinical isolates were indeed *C.albicans*, and a clear 250-bp band was observed (Fig. 2).

### *C.albicans* genotyping

**Fig. 3:**
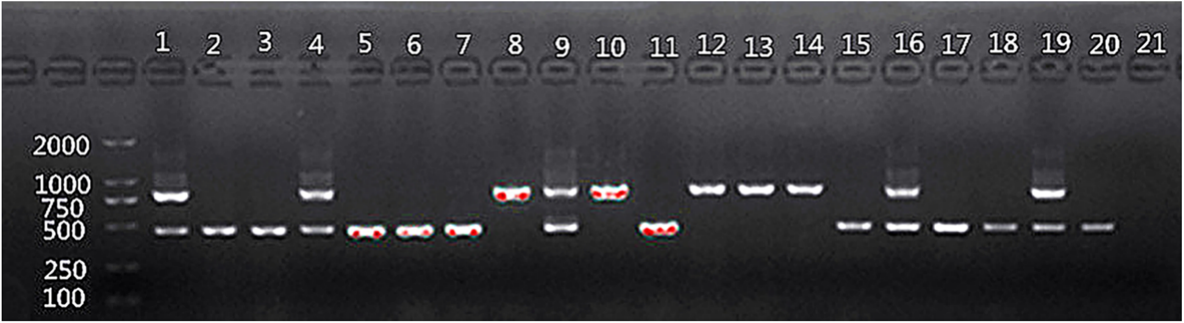
C.albicans genotyping. Lanes 2, 3, 5–7,11, 15, 17, 18, and 20: genotype A; lanes 8, 10, 12, 13, and 14: genotype B; lanes 1, 4, 9, 16, 19, and: genotype C; lane 21: negative control

The 25S rDNA PCR products were resolved on agarose gels: genotype A, 450-bp band; genotype B, 840-bp band; genotype C, 450- and 840-bp bands (Fig. 3). It revealed that genotype A was dominant in both Han and Uygur children with ECC, but the constituent ratio did not differ significantly. In Uygur CF children, genotype C was dominant, and the distribution between ECC and CF groups significantly differed. A, B, and C: *P* = 0. 2, 0.407, and 0.022, respectively; the differences in A-, B-, and C-genotype constituent ratios were not statistically significant(Table 3).

**Table 3:** Gene polymorphism of *C.albicans* isolates from Uygur and Han children with ECC or CF

Ethnicity	A-genotype (%)	B-genotype (%)	C-genotype (%)	P
Han ECC	13 (65)	4 (20)	3 (15)	0.827
Uygur ECC	11 (55)	5 (25)	4 (20)	
Uygur CF	6 (30)	2 (10)	12 (60)	
P_(Uygur ECC/CF)_	0. 2	0.407	0.022	0.035

*ECC* early childhood caries, CF caries-free

C.albicans gene polymorphism in Uygur and Han children with ECC: *n* = 40; theoretical frequency 1 ≦ T ≦ 5; Fisher’s exact test, *P* = 0.827

C.albicans gene polymorphism in ECC and CF groups of Uygur children: *n* = 40; theoretical frequency 1 ≦ T ≦ 5; Fisher’s exact test, *P* = 0.035

### The acidogenicity, aciduricity, and adhesiveness of *C. albicans*

The acidogenicity of *C.albicans* isolated from ECC and CF groups of Uygur children increased with increasing glucose concentration in the medium. When the concentration of glucose reached 0. 1 mol·L^-,1^ both the acidogenicity and growth of *C.albicans* were more pronounced in ECC group than in CF; the differences were statistically significant (acidogenicity, *P* = 0.004; growth, *P* = 0.012). *C.albicans* isolates were still alive at pH 4.0 and 4. 5; the acidogenicity and aciduricity of isolates from the ECC group were higher than the isolates from the CF group (pH 4.0, *P* = 0.012; pH 4. 5, *P* = 0.029). Finally, the average glass-wall adhesion ratio was higher in the isolates from the ECC group (53.92%± 6.79%) than in isolates from the CF group (31. 12%± 5.45%, *P* = 0.02).

### *C.albicans* sap activity

As determined by the YNB-BSA-agar method, all strains showed Sap activity. The activity was significantly higher in ECC group isolates (0.160 ± 0.012) than in CF group (0.217 ± 0.031; *t* = 7.713, *P* < 0.001). Similarly, based on the results of the MTT assay, *C.albicans* Sap activity was significantly higher in ECC group isolates (1.876 ± 0.373) than in CF group(1.166 ± 0.348; *t*= 6.226, *P* < 0.001).

### *SAP1–*5 gene expression

The *SAP1–*3 and *SAP5* genes were expressed at higher levels in ECC group than in CF, but the difference in expression was only statistically significant for *SAP2* (*P* < 0.001) and *SAP5* (*P* = 0.001) expression; conversely, *SAP4* expression was lower in ECC group than in CF, but the difference was not significant (*P* = 0.114).

### Cariogenicity of *C. albicans* of different genotypes: Factorial analysis

By using 3× 2× 2 factorial analysis, at pH 4.0 and 4. 5 the aciduricity did not differ between the different genotypes, but was higher in the ECC group than in the CF group isolates.

Acidogenicity *C.albicans* of different genotypes from ECC and CF groups in the presence of 0. 1 mol·L^-1^ of glucose was compared using 3× 2 factorial analysis; it significantly differed between the groups (group comparison, *P* = 0.020; genotype comparison, *P* = 0.019); ECC group: A > C > B; CF group: B > A > C(Table 4).

**Table 4:** Acidogenicity of A-, B-, and C-genotype *C.albicans* isolates from the ECC and CF groups in the presence of 0. 1 mol·L-1 glucose

Genotype	ECC	CF	P
A	3.092 ± 0.230	2.772 ± 0.125	0.019
B	2.656 ± 0.308	2.825 ± 0.078	
C	2.870 ± 0.424	2.617 ± 0.193	
P	0.020		

*ECC* early childhood caries, CF caries-free

ECC group: A > C > B; CF group: B > A > C

**Fig. 4:**
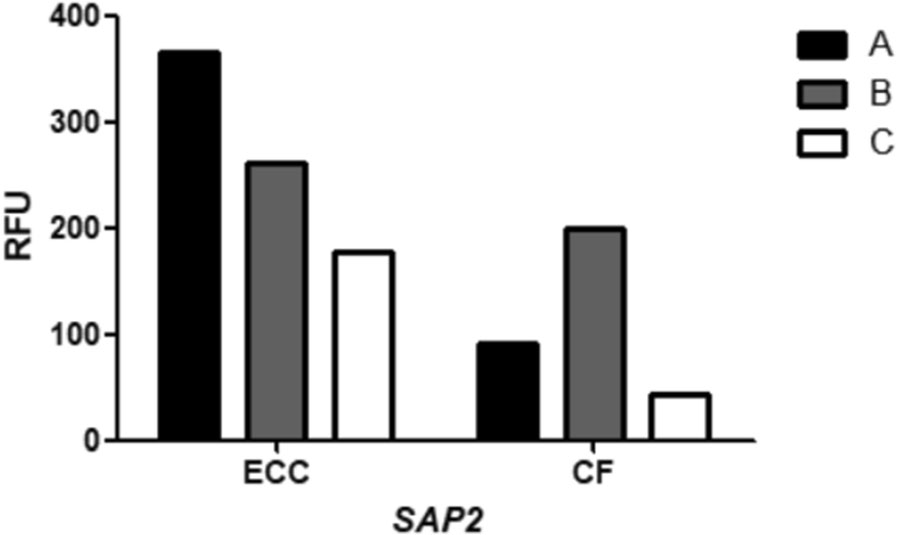
*SAP2* expression in A-, B-, and C-genotype *C.albicans*: differences between the ECC and CF group isolates

*C. albicans* adhesion rate and Sap activity were examined using 3× 2 factorial analysis, which revealed that these did not show genotype-dependent differences. Finally, factorial analysis of gene expression in ECC and CF group isolates revealed that only the expression of *SAP2* was significantly different in *C.albicans* of different genotypes in the ECC and CF groups (group comparison, *P* = 0.001; genotype comparison, *P* = 0.020); *SAP2* expression: ECC group, A > B > C; CF group, B > A > C (Table 5,Fig. 4).

**Table 5:** SAP2 expression in A-, B-, and C-genotype *C. albicans*: differences between the ECC and CF group isolates

Genotype	ECC	CF	P
A	3.644± 1.415	0.910 ± 0.897	0.020
B	2.617± 1.481	1.995 ± 0.332	
C	1.783± 1.373	0.435 ± 0.652	
P	0.001		

*ECC* early childhood caries, CF caries-free

SAP2 expression: ECC group, A > B > C; CF group, B > A > C

## Discussion

The recent research into the aetiology of ECC has been focused on the relationship between *C.albicans* and ECC [16]. Klinke et al. verified that *C.albicans* is associated with an increased prevalence of caries [17]. In the current study, the *C.albicans* detection rate in the ECC group (48.8%) was more than double of that in the CF group (23.8%), which again demonstrated the close association of *C.albicans* with ECC.

The *C.albicans* detection rate was significantly higher in Uygur children than in Han (*P* = 0.009), in contrast to our previous study of Urumqi [18]. This discrepancy might be due to regional differences: compared with Urumqi, Kashgar is a more isolated city, with a higher proportion of Uygur people and unique customs. Hence, *C.albicans* distribution is likely affected not only by ethnic differences, but also by geographic differences.

We found that in the presence of 0. 1 mol·L^-1^ of glucose, the acid produced by *C.albicans* caused the broth pH to drop from 7.00 to 4.06, which is lower than the enamel demineralization pH threshold (5. 5) [19]; further, *C.albicans* acidogenicity and growth were both more pronounced in ECC group isolates than in CF group. This suggests that *C.albicans* isolates were strongly cariogenic in the presence of 0. 1 mol·L^-1^ of glucose. Furthermore, the aciduricity and growth capacity of both ECC and CF group *C.albicans* isolates decreased as the initial pH was lowered; nevertheless, at pH 4.0, *C.albicans* continued to produce acid, in agreement with the observations of Klinke et al. [18] This is in contrast with clinical isolates of *S.mutans*, whose growth is inhibited, and acidogenicity and aciduricity are abolished at pH below 5.0 [20], indicating that in an acidic environment, the acidogenicity and aciduricity of *C.albicans* are more pronounced than in *S.mutans*.

*C.albicans* is able to colonize the oral cavity because of its strong adhesion ability. The activity of *C.albicans* Saps has been shown to be associated with *C.albicans* invasiveness, and cell adhesion is proportional to the amount of produced Saps [21]. Recently, considerable progress has been made in the *C.albicans* Sap research. Taylor et al. reported that Sap5 is expressed early in infection; Sap4 is expressed in the final infection stage, while the expression of Sap5 is reduced [22]. The difference in Sap expression is linked to the infection environment [23], and this might due to the adaptation of Sap to the specific host environment, promoting an overlap in the activities of distinct Sap proteins produced in diverse environments and their roles. The results of the current study differ from those of Li et al. [24], who reported significantly higher or lower expression of *SAP1* and *SAP4* respectively in ECC group isolates than in CF group. This discrepancy might be associated with the differences between the selected populations, geographical differences, or ethnic differences, but further investigation is required to clarify this issue. In the future, DNA-chip technology and microarray analysis may allow the elucidation of the relationship between the expression of individual *SAP* genes and the biological activity or virulence of *C.albicans*, facilitating the development of new infection prevention strategies based on targeting of specific *SAP* genes.

Genotyping is an effective and classical molecular biology approach for studying the relationship between species, clarifying the association between different strains. The genotype of *C.albicans* continually changes after host infection to enable fungal adaptation to new environments [25]. In this regard, the transmission and colonization of oral microorganisms is not only limited by the source and route of infection, but is also affected by the host’s genetic background, lifestyle, and oral microbial environment [26].The constitution of genotype A in the current study is the same as reported by Wu et al. [10], and da Silva-Rocha et al. [27], implying that the *C.albicans* of genotype A might cause more caries. Genotype C of *C.albicans* was dominant in Uygur CF children, and the difference between Uygur ECC and CF group isolates was statistically significant. This results differ from those of Qiu et al [28], possibly on account of location differences, and the unique eating habits and lifestyles of the subjects. *C.albicans* exhibits extremely high gene polymorphism in hosts from different ethnic groups and in hosts with different degree of caries [[10], [18]] this suggests that more attention should be devoted to genotype variation and host microecological balance when addressing such topics as *C.albicans* pathogenicity, prevention of dysbacteriosis, and drug selection. In the current study, the constituent *C.albicans* genotype was different, probably because of differences in pathogenicity, or selective growth of *C.albicans* in different host environments. The following conclusion may hence be drawn pertaining to the differences in genotype constitution: the mouth micro-environment differs among different groups of people, and thus the colonization capacity of different genotypes of *C.albicans* is also different.

In conclusion, dental caries develops when the dynamic equilibrium of the oral microflora is destroyed. The results might be correlate with the differences in population genetics and ethnicity, geographical distribution, and eating habits. Further investigation is required to verify that. To fully understand the ecological processes of caries formation, future molecular biology research is essential: sophisticated methods, such as metabolomics and metagenomics, are needed for the reconstruction of microbial metabolism networks and the dynamic stability of physiological mechanisms within a dental biofilm.

## Conclusions

The strong acidogenicity and aciduricity, Sap activity, and high SAP2 and SAP5 expression might be closely associated with ECC. C.albicans potentially plays a key role in the progression of caries, which most readily affects genotype A carriers and could be attributed to person-to-person environmental variation.

## Abbreviations

BSA: Bovine Serum Albumin
CAC: Chromagar Candida
ECC: Early childhood caries
MTT: Methy thiazolyl tetrazolium
PCR: Polymerase Chain Reaction
Sap: Secreted aspartyl proteinase
SDA: Sabourand Dextrose Agar
YNB: Yeast Nitrogen Base
